# Chemical compound-based direct reprogramming for future clinical applications

**DOI:** 10.1042/BSR20171650

**Published:** 2018-05-08

**Authors:** Yukimasa Takeda, Yoshinori Harada, Toshikazu Yoshikawa, Ping Dai

**Affiliations:** 1 Department of Cellular Regenerative Medicine, Graduate School of Medical Science, Kyoto Prefectural University of Medicine, 465 Kajii-cho, Kawaramachi-Hirokoji, Kamigyo-ku, Kyoto 602-8566, Japan; 2 Department of Pathology and Cell Regulation, Graduate School of Medical Science, Kyoto Prefectural University of Medicine, 465 Kajii-cho, Kawaramachi-Hirokoji, Kamigyo-ku, Kyoto 602-8566, Japan; 3 Louis Pasteur Center for Medical Research, 103-5 Tanaka-Monzen-cho, Sakyo-ku, Kyoto 606-8225, Japan

**Keywords:** chemical compound, direct reprogramming, regenerative medicine

## Abstract

Recent studies have revealed that a combination of chemical compounds enables direct reprogramming from one somatic cell type into another without the use of transgenes by regulating cellular signaling pathways and epigenetic modifications. The generation of induced pluripotent stem (iPS) cells generally requires virus vector-mediated expression of multiple transcription factors, which might disrupt genomic integrity and proper cell functions. The direct reprogramming is a promising alternative to rapidly prepare different cell types by bypassing the pluripotent state. Because the strategy also depends on forced expression of exogenous lineage-specific transcription factors, the direct reprogramming in a chemical compound-based manner is an ideal approach to further reduce the risk for tumorigenesis. So far, a number of reported research efforts have revealed that combinations of chemical compounds and cell-type specific medium transdifferentiate somatic cells into desired cell types including neuronal cells, glial cells, neural stem cells, brown adipocytes, cardiomyocytes, somatic progenitor cells, and pluripotent stem cells. These desired cells rapidly converted from patient-derived autologous fibroblasts can be applied for their own transplantation therapy to avoid immune rejection. However, complete chemical compound-induced conversions remain challenging particularly in adult human-derived fibroblasts compared with mouse embryonic fibroblasts (MEFs). This review summarizes up-to-date progress in each specific cell type and discusses prospects for future clinical application toward cell transplantation therapy.

## Introduction

Terminally differentiated cells acquire a pluripotent state by forced expression of Yamanaka’s reprogramming factors [1–3]. Over the last 10 years, this discovery has provided a basic technique to manipulate cell fates in a transcription factor-based manner. The induced pluripotent stem (iPS) cells can be differentiated into numerous cell types, and are expected to be applied to drug screening, disease modeling, and transplantation therapy. However, both the reprogramming and differentiation processes raised potential safety concerns for the clinical application. The exogenous gene induction for the reprogramming might cause genomic instability and mutations [4]. In addition, contamination of residual undifferentiated cells after differentiations increases the risk for undesirable tumorigenesis after transplantation [5]. Currently, the characterization of pluripotency, differentiation potential, and genomic integrity in heterogeneous colonies of iPS cells is time-consuming and expensive, so it is not feasible that somatic cells isolated from each patient can be reprogrammed into iPS cells for their own transplantation therapy to avoid immune rejection [6]. Recently it has been reported that allogenic iPS cells with the same homozygous human leukocyte antigen (HLA) haplotype as the patient still can cause immune rejection responses in *in vitro* assay system [7]. The evidence also implies the importance of the use of each set of patient-derived autologous cells for their own transplantation therapy.

To solve the problems described above, direct lineage reprogramming or transdifferentiation is a promising alternative to rapidly prepare desired cell types from somatic cells by bypassing a pluripotent state. The direct reprogramming is generally achieved by forced expression of a set of lineage-specific transcription factors to establish a transcriptional network similar to the one in the specific cell type along with change in epigenetic modifications. So far accumulating evidence has demonstrated the direct reprogramming of mouse and human dermal fibroblasts into various cell types including neurones, neural stem cells, cardiomyocytes, hepatocytes, and brown adipocytes [8–12]. These reports indicate that the cell fate conversion is more flexible than we previously assumed. If desired cell types are rapidly converted within several weeks, autologous fibroblasts can be used for the patients’ own transplantation therapy. However, due to the requirement of simultaneous expression of multiple transcription factors in a single cell, the efficiency of the direct reprogramming is generally insufficient for the use of transplantation therapy without any sorting or purification steps. In addition, exogenous gene induction unexpectedly raises the risk for genomic instability and mutations, which might lead to tumor formation. The directly reprogrammed cells might be engrafted over a few years after transplantation to compensate for deficiency of tissue functions. Therefore, such a risk for tumorigenesis needs to be the lowest for the clinical applications to the maximum extent possible.

**Figure 1:**
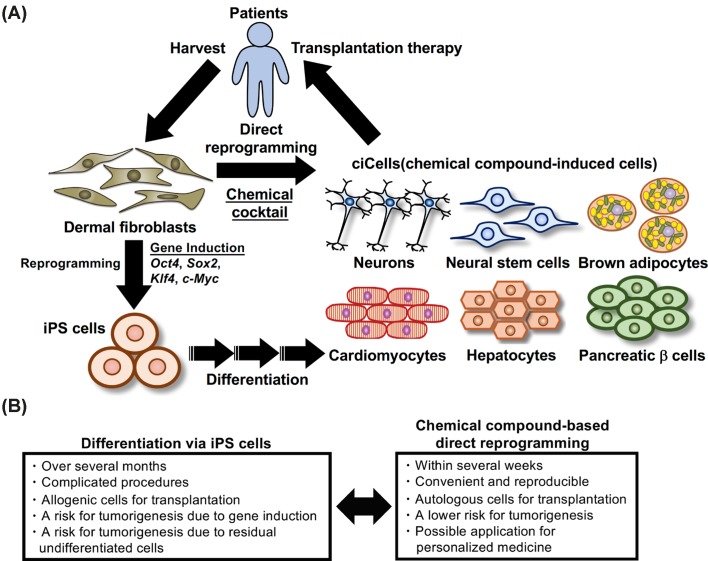
Schematic figure for chemical compound-based direct reprogramming of human dermal fibroblasts into desired cell types applicable for transplantation therapy (**A**) Human dermal fibroblasts isolated from each patient are chemically converted into several desired cell types including neurones, neural stem/progenitor cells, brown adipocytes, cardiomyocytes, hepatocytes, and pancreatic β cells by regulating cellular signaling pathways and histone/DNA modification enzymes. iPS cells are reprogrammed by forced expression of transcription factors, then differentiated into these cells. (**B**) Compared with the differentiation via iPS cells, chemical compound-based direct reprogramming has a number of clear advantages for transplantation therapy, disease modeling, and drug development. In particular, the derivation and characterization of iPS cells for clinical applications still takes a long time and is expensive, so the use of autologous iPS cells derived from each patient might not be feasible. Chemical compound-based direct reprogramming is expected to rapidly convert autologous fibroblasts into desired cell types for the patients’ own transplantation. In addition, the chemically converted cells might have a lower risk for tumorigenesis compared with the cells prepared by forced expression of transcription factors as well as the cells inefficiently differentiated from iPS cells.

Recently, significant progress has been made in the field of direct lineage reprogramming by means of chemical compounds alone. The cell fate conversions are performed without the use of transgenes by regulating cellular signaling pathways and activity of histone/DNA modifying enzymes. In the beginning, scientists discovered a number of small molecules which significantly facilitate somatic cell reprogramming into iPS cells. Some of them enable replacement effects of the reprogramming factors such as *Oct4*, *Sox2*, and *Klf4* [13–15]. Small molecules also can promote the efficiency of transcription factor-based direct reprogramming and sometimes replace the effects of transcription factors and cytokines, which is obviously helpful in preparing a large amount of cells in a defined and cost-effective manner [16,17]. They have several unique advantages in that they are preserved, highly purified, have a long half-life, are non-immunogenic, and effective at a low concentration. Accumulating evidence has demonstrated that mouse embryonic fibroblasts (MEFs) and/or human dermal fibroblasts were converted into several useful cell types including neurones, astrocytes, neural stem cells, brown adipocytes, cardiomyocytes, endoderm progenitor cells, and pluripotent stem cells (Figure 1A). However, in many cases the direct conversion is still particularly challenging in adult human fibroblasts compared with MEFs. In addition, complete chemical compound-based direct conversion into endoderm lineage cells such as hepatocytes and pancreatic β cells, the most important cell types in current regenerative medicine, has not been reported yet. Chemical compounds potentially affect wide-range gene expression and epigenetic modifications compared with the ones regulated by specific transcription factors. Nevertheless, the chemical compound-based strategy does not involve forced expression of exogenous genes as well as a pluripotent state, which could provide better cell sources for clinical applications with the lower risk for tumorigenesis (Figure 1B). This review summarizes the recent progress on chemical compound-based direct reprogramming in each specific cell type, problems to be solved, and future prospects for clinical applications.

## Neuronal cells

It is not feasible to prepare a sufficient amount of primary human neuronal cells to study neurodegenerative disorders such as Alzheimer’s disease, Parkinson’s disease, and amyotrophic lateral sclerosis (ALS). In 2010, Vierbuchen et al. [8] first reported that MEFs were converted into neurones by forced expression of neurone-specific transcription factors, Brn2, Ascl1, and Myt1 (BAM). Later, a number of groups reported direct conversion of mouse and human fibroblasts into different neurone subtypes such as dopaminergic neurones and motor neurones by combination of BAM and each set of subtype-specific transcription factors [18–20]. These studies provided numerous insights into development of neural direct reprogramming in a chemical compound-based manner.

We first reported that the combination of six chemical compounds rapidly converted human dermal fibroblasts into chemical compound-induced neuronal cells (CiNCs) (Table 1) [21,22]. It should be noted that neonatal and adult dermal fibroblasts with different ages and genders were all converted efficiently. Not only a neuronal marker, Tuj1, but also the mature markers, MAP2 and Synapsin, were expressed in CiNCs. CiNCs were positive for both vGlut1 and GABA, suggesting that both glutamatergic (excitatory) and GABAergic (inhibitory) neurones were likely mixed in the population. The heterogeneous population of excitatory and inhibitory neurones shows the same tendency as reported in the other direct reprogramming into neuronal cells [8]. CiNCs exhibit a spike-like neuronal activity (unpublished data), suggesting that CiNCs could be functional neurones with basic electrophysiological properties. A spinal cord injury requires a rapid transplantation of neuronal cells at the subacute phase for efficient engraftment and recovery [23]. CiNCs can be rapidly prepared from autologous fibroblasts, and the risk for tumorigenesis is considered to be lower. Therefore, the transplantation of CiNCs might be one of the most promising strategies not only for treatment of spinal cord injury but also for other clinical applications. In addition, other neuronal subtypes such as cholinergic, serotonergic, and dopaminergic neurones might be generated by modifying the combination of chemical compounds, growth factors, and culture protocols.

**Table 1:** Summary of chemical compound-based direct reprogramming into neurones

Target cell type	Source cells	Generation	Chemical cocktail and cytokines	Induction/maturation medium	References
Neurone (glutamatergic neurones and GABAergic neurones)	Human dermal fibroblasts	Neonatal and adult	CHIR99021, PD0325901, LDN193189, SB431542, Pifithrin-α, and Forskolin	The N2B27 medium with the chemical cocktail for several weeks	[21]
Neurone (glutamatergic neurones and GABAergic neurones)	MEFs	Fetal	Forskolin, ISX9, CHIR99021, and I-BET151	The neuronal induction medium with the chemical cocktail and basic fibroblast growth factor (bFGF) for 20 days. The maturation medium in co-culture of primary astrocytes or the neuronal induction medium containing Forskolin, bFGF, BDNF, GFNF for another 15 days	[24]
Neurone (glutamatergic neurones and GABAergic neurones)	Human foreskin fibroblasts, human dermal fibroblasts	Adult	VCRFSGY: VPA, CHIR99021, RepSox, Forskolin, SP600125, GO6983, and Y-27632	The induction medium with the chemical cocktail for 8 days. The maturation medium with CHIR99021, Forskolin, and Dorsomorphin, and extra neurotrophic factors, BDNF, GDNF, and NT3 for another 6 days	[25]
Neurones (glutamatergic neurones)	Human primary astrocytes	Fetal	LDN193189, SB431542, TTNPB, Thiazovivin, CHIR99021, VPA, DAPT, SAG, and Purmophamine	The N2 medium with the nine chemical compounds was sequentially treated in a stepwise manner for 8 days. The neuronal differentiation medium containing three neurotrophic factors, BDNF, NT3, and IGF-1 from day 9 to 30 for the maturation	[30]
Neurones (glutamatergic neurones)	Human primary astrocytes	Adult	VPA, CHIR99021, RepSox, Forskolin, I-BET151, and ISX-9	The induction medium with the chemical cocktail for 20 days. The neurone maturation medium with Forskolin and I-BET151 for further culture	[32]
Astrocytes	MEFs, human foreskin fibroblasts	Fetal	VAP, CHIR99021, SB431542, Tranylcypromine, and OAC1	The induced astrocyte medium with fibroblast growth factor 2 (FGF2) and the chemical cocktail for 25 days (mouse) or 40 days (human)	[33]

Thereafter, other groups also showed that MEFs and human dermal fibroblasts were, respectively, converted into functional neurones by a different set of chemical compounds. MEFs were converted into chemically induced neurones by four compounds including ISX9, Forskolin, CHIR99021, and I-BET151 [24]. A BET family Bromodomain inhibitor, I-BET151, repressed fibroblast specific gene expression to facilitate the direct conversion, while ISX9 is required for activation of neurone-specific gene expression. The chemically induced neurones are a mixture of glutamatergic and GABAergic neurones. Further study is required to know how the chemical combination is beneficial in the conversion of human dermal fibroblasts. Human adult fibroblasts derived from healthy individuals as well as familial Alzheimer’s disease patients were converted into neurones by seven chemical compounds, VCRFSGY, including VPA, CHIR99021, RepSox, Forskolin, SP600125, GO6983, and Y-27632 [25]. This group previously reported a conversion of MEFs into neural progenitor cells (NPCs) by combination of the VCR and hypoxia [26]. According to their hypothesis, the combination of chemicals for neuronal differentiation (FSGY) and partial reprogramming into NPCs (VCR) successfully converted human fibroblasts into neurone-like cells. After the induction of neural reprogramming, subsequent treatment with CHIR99021, Forskolin, and Dorsomorphin and extra neurotropic factors, BDNF, GDNF, and NT3, further promoted neuronal maturation and improved cell survival. The chemically induced neurones were also a mixture of glutamatergic and GABAergic neurones. The authors suggested that other neuronal subtypes might be generated with slightly modified chemical cocktails. The chemical direct reprogramming could be applied to generate patient-specific neuronal cells for neurological disease modeling, related mechanism studying, and drug screening. The amyloid β levels of the chemically induced neuronal cells were similar to those of transcription factor-induced neuronal cells derived from each familial Alzheimer’s disease patient, which suggests that chemical direct reprogramming provides an alternative approach for personalized disease modeling.

Meanwhile, several groups reported that neurones were directly converted from astrocytes by overexpression of exogenous transcription factors both *in vitro* and *in vivo* [27–29]. Astrocytes can be a cell source to regenerate neurones directly in the brain without transplantation. Zhang et al. [30] reported that sequential exposure of a set of chemical compounds directly converted primary astrocytes isolated from human fetal brain into neurones. The nine compounds used in the four steps are LDN193189, SB431542, TTNPB, Thiazovivin, CHIR99021, VPA, DAPT, SAG, and Purmophamine. Most of the astrocyte-derived neurones were glutamatergic neurones, and negative for the markers of cholinergic neurones, dopaminergic neurones, and spinal motor neurones. The neurones transplanted into mouse brain were engrafted over 1 month. Their protocol is available for human astrocytes but not mouse astrocytes, and for brain astrocytes but not spinal cord astrocytes, suggesting that the effects of chemical compounds and molecular mechanism underlying the direct reprogramming are slightly different in species and cellular subtypes. The transgene-free chemical-based technique might be further developed to regenerate neurones from astrocytes in a particular brain region *in vivo*. For clinical application of the *in vivo* reprogramming, a simpler protocol for treatment with the compounds and methods to deliver them into a particular brain region are required.

Human fetal astrocytes chemically converted into neurones, as described above, have different functional properties from adult astrocytes [31]. For the clinical application of *in vivo* direct reprogramming, adult astrocytes also need to be converted into neurones. Recently, Yu et al. [32] demonstrated that human adult astrocytes were more simply converted into neuronal cells by a different set of chemical compounds including VPA, CHIR99021, RepSox, Forskolin, I-BET151, and ISX-9. Tian et al. [33] reported that both mouse and human fibroblasts are directly converted into astrocytes in a chemical compound-based manner. The chemically induced astrocytes resemble primary astrocytes in terms of gene expression and functional properties. These reports expand a strategy to manipulate cell fates in fibroblasts, astrocytes, and neurones by using chemical compounds only.

**Figure 2:**
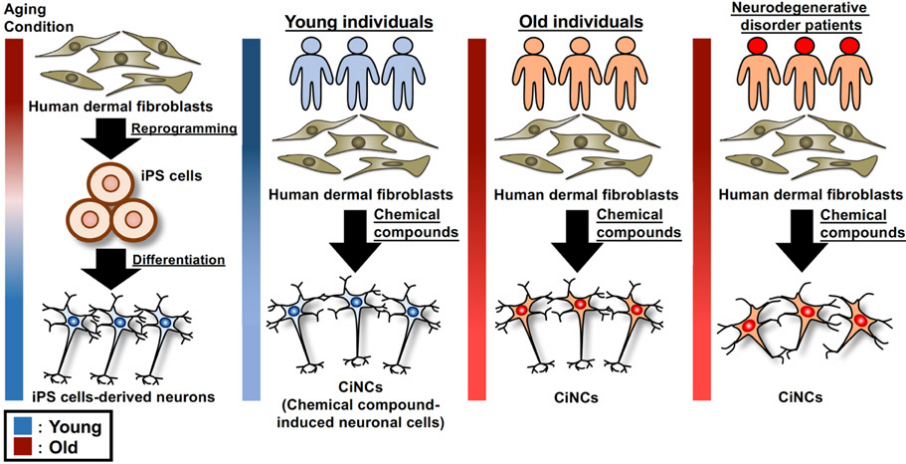
Schematic figure for a possible application of chemical compound-induced neurones to uncover age-associated pathological mechanism The CiNCs are generated from human dermal fibroblasts isolated from young individuals, old individuals, and neurodegenerative disorder patients, respectively. The CiNCs might conserve donor-dependent ageing conditions, while it has been reported that iPS cells derived from the fibroblasts do not retain the ageing conditions. Comparison of transcriptome and repressive histone modifications in the induced neurones derived from young and old individuals might lead to identifying novel age-associated gene targets and the pathological mechanism underlying neurodegenerative disorders.

Along with increasing human longevity, the number of patients with neurological disorders is increasing. Ageing is one of the major risk factors for neurodegenerative disorders which are generally caused by abnormal accumulation of misfolded proteins in specific neurone subtypes [34,35]. It has been reported that the reprogramming into iPS cells by forced expression of Yamanaka’s factors erases most of the age-associated cellular and epigenetic marks [36]. To study a relation between ageing and neurological disorders, neuronal cells differentiated from iPS cell lines might not be appropriate as a disease model. The advantage of direct reprogramming is bypassing a pluripotent state, so the induced neuronal cells might conserve the same ageing conditions as source cells. Mertens et al. [37] demonstrated that neurones induced from human fibroblasts by transcription factors retained donor-age-dependent transcriptomic signatures and showed age-associated defects of nucleocytoplasmic compartmentalization. This observation implies that such ageing conditions might be conserved also in chemically induced neurones (Figure 2). Because forced expression of exogenous transcription factors for the direct reprogramming is supposed to unexpectedly damage proper epigenetic marks and genome integrity, such stress of chemical compound-based conversion should be milder, leading to better conservation of the ageing conditions. In the near future, the transgene-free, chemical-based strategy might be applied for personalized disease modeling to uncover the age-related pathological mechanism underlying neurodegenerative disorders.

## Neural stem cells

Cheng et al. [26] first reported direct conversion of MEFs, tail-tip fibroblasts, and human urinary cells into NPCs by a chemical cocktail consisting of VPA, CHIR99021, and RepSox under physiological hypoxic condition (5% O_2_) (Table 2). After the induction, the lineage specification in the chemically induced NPCs was gradually promoted by passaging in the neural expansion medium containing epidermal growth factor (EGF) and basic fibroblast growth factor (bFGF). The NPCs expressed major neural stem cell marker genes, *Sox2* and *Nestin*, and could be differentiated into neurones, astrocytes, and oligodendrocytes. These results indicate that the induced NPCs resemble typical neural stem cells. A physiological hypoxia condition is likely beneficial in both the induction of NPCs and the proliferation. Such a hypoxic condition has been known to promote the reprogramming efficiency from fibroblasts into iPS cells by changing metabolic switch from oxidative to glycolytic metabolism [38]. In addition, the hypoxia condition might facilitate the conversion by activating the WNT signaling pathway, which is also activated by CHIR99021, an inhibitor of glycogen synthase kinase 3β (GSK3β) [39].

**Table 2:** Summary of chemical compound-based direct reprogramming into neuronal stem/progenitor cells

Target cell type	Source cells	Generation	Chemical cocktail and cytokines	Induction/maturation medium	References
NPCs	MEFs, mouse tail-tip fibroblasts, human urinary cells	Fetal neonatal adult	VPA, CHIR99021, and RepSox	The KSR medium (15% KSR) with the chemical cocktail for 10 days under 5% O_2_ condition	[26]
Neural stem cells	MEFs. mouse tail-tip fibroblasts	Fetal adult	VPA, BIX01294, RG108, PD0325901, CHIR99021, vitamin C, and A83-01	The stem cell culture medium with the chemical cocktail for 1 day, then the medium without the compounds for 2 days. This cycle was repeated for a total of six times. The cells were cultured in the neural stem cell medium by suspending culture for another 2 weeks	[40]
Neural stem cells	MEFs	Fetal	A83-01, Thiazovivin, Purmophamine, and VPA	The standard NSC medium with the chemical cocktail for 10–12 days	[41]
Neural stem cells	MEFs	Fetal	CHIR99021, LDN193189, A83-01, RG108, Parnate, SMER28, retinoic acid, Hh-Ag1.5, and bFGF	The neural reprogramming basal medium with the chemical cocktail for 14 days under 5% O_2_ condition. After picking up colonies, the NSC medium under normal O_2_ condition for further culture	[42]
Neural stem cells	Human adipose-derived mesenchymal stem cells	Adult (23–26 years old)	SB431542, Noggin, and LDN193289	The NSC medium containing the chemical cocktail and 3% KSR for 8 days. The B27N2 medium without EGF and fibroblast growth factor 2 (FGF2) for another 5 days. The B27N2 medium with EGF and FGF2 for another 7 days	[43]
Neural stem cells	Primary murine astrocytes	Adult	bFGF	The N2B27 medium including bFGF for 8 days	[44]

Han et al. [40] reported direct reprogramming of MEFs into neural stem cells by using chemical compounds only. The culture protocol is uniquely repeating change of the medium with or without the chemical cocktail containing VPA, BIX01294, RG108, PD0325901, CHIR99021, vitamin C, and A83-01 for a total of six times. Then the 2-week suspension culture formed neurospheres expressed typical neural stem cell markers. The treatment with either chemical compounds such as A83-01, Thiazovivin, Purmophamine, VPA, or combinations of their substituents also induced the direct reprogramming from MEFs into neural stem cell-like cells [41]. The chemically induced neural stem cells described in these reports were tripotent and differentiated into neurones, astrocytes, and oligodendrocytes. Several common and different compounds were utilized in these various studies, so further studies are required to elucidate how signaling pathways regulated by each of them enable the direct conversion of MEFs into neural stem cells.

A different set of eight chemical compounds including CHIR99021, LDN193189, A83-01, RG108, Parnate, SMER28, retinoic acid, Hh-Ag1.5, and a growth factor, bFGF, was used for the direct reprogramming of MEFs into neural stem cells [42]. By genetic lineage tracing, the authors clearly demonstrated that the fibroblasts were converted into neural stem cells. They also identified signaling pathways and downstream transcription factors required for the direct conversion. They are Elk1 and Gli2 which are located at the downstream of the mitogen-activated protein kinase (MAPK) signaling pathway activated by bFGF and Sonic hedgehog (SHH) signaling pathway activated by Hh-Ag1.5, respectively. They were both recruited to the promoter region of *Sox2*, a master transcription factor for neural stem cells. The inhibition of these signaling pathways with other chemical compounds greatly reduced the reprogramming efficiency, suggesting that the activation of MAPK and SHH signaling pathways plays a pivotal role in the chemical direct reprogramming into neural stem cells. The present study provides great insights into the molecular mechanism underlying chemical induction of neural stem cells from mouse fibroblasts.

Most of these studies converted MEFs into neural stem cells, but for the clinical application, human adult fibroblasts have to be converted in a chemical compound-based manner. MEFs might have a relatively higher differentiation potential and plasticity than adult fibroblasts. Moreover, gene expression regulated by each chemical compound through signaling pathways and downstream transcription factors is supposed to be slightly different between mouse and human fibroblasts. Nevertheless, the identification of chemical compounds required for the conversion into neural stem/progenitor cells helps us further develop a combination of chemical compounds for human adult fibroblasts. The next step is to identify the combination showing a broad utility to the direct conversion in human fibroblasts. The chemically induced neural stem cell is a promising cell source for future transplantation therapy to treat neural injuries and degenerative diseases.

Mesenchymal stem cells (MSCs) are another cell source for direct reprogramming or transdifferentiation induced by chemical compounds. Previously, several reports have demonstrated that neural stem cell-like cells were converted from MSCs by culture medium including growth factors and chemical compounds without the use of any transgene, but the reproducibility and efficiency appeared not to be always sufficient. Recently, human adipose-derived MSCs were converted into neural stem cells by using SB431542, Noggin, and LDN193289, which are inhibitors of transforming growth factor β (TGFβ) and bone morphogenetic protein (BMP) signaling pathways [43]. This affords an efficient generation of functionally induced neural stem cells, and can be a cell source for transplantation therapy. Neural stem cells were also induced from mouse ES cell-derived astrocytes as well as primary astrocytes by a treatment with fibroblast growth factor 2 (FGF2) for 8 days [44]. The treatment with FGF2 promoted both proliferation and expression of Nestin, a neural stem cell marker, in the astrocytes, suggesting that downstream signaling pathways regulated by FGF2 play a critical role in the induction of neural stem cells.

## Brown adipocytes

Obesity is a major risk concern for metabolic diseases including diabetes and cardiovascular diseases. Previous studies have revealed that brown adipocytes in our body also play an important role in physiological regulation of energy metabolism and heat production, which leads to prevention of obesity. Interestingly, it has been demonstrated that white adipocytes can be transdifferentiated into brown adipocytes, so-called beige adipocytes, to consume the storing lipid [45]. It still remains controversial whether white adipocytes or specific progenitor cells are changed into beige adipocytes *in vivo* [46]. So the direct reprogramming into brown adipocytes is an interesting approach to uncover the molecular mechanism underlying the transdifferentiation and proliferation in our body.

The traditional brown adipocytes are differentiated from myogenic precursor cells expressing *Myf5* [47]. Recent studies have reported that functional brown adipocytes were prepared from iPS cells, MSCs, and myoblasts in a transcription factor-based or growth factor-based manner [48–50]. In 2009, Kajimura et al. [12] reported that forced expression of PRDM16 and C/EBP-β converted MEFs to induced brown adipocyte-like cells. Recently, human dermal fibroblasts were converted into brown adipocytes by forced expression of two transcription factors, C/EBP-β and C-MYC [51]. For the clinical application, a novel strategy without the use of transgenes had been anticipated to prepare brown adipocytes from human dermal fibroblasts.

Chemical compound-based direct reprogramming into brown adipocytes is a useful strategy to reveal how brown fat mass is chemically increased in our body. Recently, Nie et al. [52] reported that Bexarotene (Bex), a retinoid X receptor (RXR) agonist, directly converted myoblasts, one of the precursor cell types, into brown-like adipocytes (Table 3). Screening of 20000 chemicals in C2C12 cells, mouse skeletal myoblasts, identified that Bex treatment at 10 μM for 2 days induced brown adipogenic reprogramming. Oral administration of Bex into mice for 4 weeks along with feeding of a high-fat diet resulted in a slight increase in brown fat mass, which is associated with prevention of weight gain, increase in oxygen consumption rate, glucose/insulin tolerance, and cold tolerance. In addition, increase in brown adipocyte cell number and expression of the specific genes were observed in subcutaneous white adipose tissue (WAT), suggesting that Bex has a browning effect through RXR activation.

**Table 3:** Summary of chemical compound-based direct reprogramming into brown adipocytes and cardiomyocytes

Target cell type	Source cells	Generation	Chemical cocktail cytokines	Induction/maturation medium	References
Brown adipocyte	C2C12 cells (mouse skeletal myoblasts)	-	Bex	The basal adipogenesis medium with Bex for 2 days. The culture under basal adipogenesis conditions for another 4 days	[52]
Brown adipocyte	Human dermal fibroblasts	Adult (0, 38, and 49 years old)	SB431542, LDN193189, Forskolin, Dorsomorphin, and Rosiglitazone	The commercial adipocyte medium with the chemical cocktail for 3 weeks. The medium without the compounds, except for Rosiglitazone, for another 1 week	[53]
Cardiomyocyte	MEFs	Fetal	CHIR99021, RepSox Forskolin, VPA, Parnate, and TTNPB	The Cardiac Reprogramming Medium (CRM) with the chemical cocktail for 16 days. The Cardiomyocyte-maintaining medium (CMM) with 2i (CHIR99021 and PD0325901), leukemia inhibitory factor (LIF), and insulin for another 8 days	[63]
Cardiomyocyte	Human foreskin fibroblasts, human fetal lung fibroblasts	Neonatal fetal	CHIR99021, A83-01, BIX01294, AS8351, SC1, Y-27632, OAC2, SU16F, and JNJ10198409	The chemical reprogramming medium (CRM) with the chemical cocktail for 6 days. The cardiac induction medium (CIM) for another 5 days. The conditioned medium incubated with H9 hESCs-derived cardiomyocytes for another 19 days	[65]

**Figure 3:**
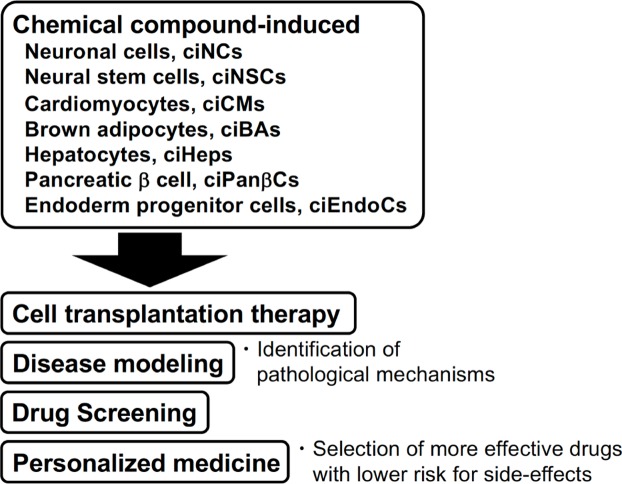
Possible applications of chemical compound-induced cells Chemical compound-induced cells (ciCells) rapidly prepared from autologous dermal fibroblasts can be a primary source for cell transplantation therapy. At the preclinical phase, the engraftment and tumorigenicity of ciCells need to be confirmed by using animal models for disease and injury. ciCells derived from each patient would be available as disease models to identify pathological mechanisms. Personalized medicine could be performed to select more effective drugs with lower risk for side effects using various kinds of ciCells. Thus, ciCells have a number of clear advantages for the clinical applications.

We recently reported that human dermal fibroblasts were converted into chemical compound-induced brown adipocytes (ciBAs) [53]. Commercially available adipocyte medium in combination with the chemical cocktail consisting of SB431542, LDN193189, Dorsomorphin, Forskolin, and Rosiglitazone for 3–4 weeks efficiently converted human dermal fibroblasts into brown adipocytes (Figure 3). It should be noted that the chemical conversion is not dependent on age, gender, and isolation sites of the fibroblasts. ciBAs were sensitive to β-adrenergic receptor signaling activated by treatment with either isoproterenol or Forskolin, and showed elevated oxygen consumption rates. In the present study, the activation of WNT signaling pathway by CHIR99021 strongly inhibited the brown adipogenesis. According to a previous report [54], TGFβ signaling pathway negatively regulates adipogenesis, so the inhibition by SB431542 facilitated the direct conversion. The inhibition of BMP signaling pathways by LDN193189 and Dorsomorphin could be involved in the modulation of downstream Smad protein activities in collaboration with the TGFβ signaling pathway. Both peroxisome proliferator-activated receptor γ (PPARγ) activation by Rosiglitazone and cAMP induction by Forskolin play an important role in brown adipogenesis and the functional induction. The accumulation of these lines of evidence would lead to better strategies for the direct conversion and differentiation of brown adipocytes by chemical compounds. The ciBAs might be applied for not only transplantation therapy but also drug development to prevent obesity and metabolic diseases by activation of energy metabolism.

*In vivo* induction of brown adipogenic reprogramming has been performed by delivering browning agents using a microneedle-based patch [55]. Nanoparticles encapsulating Rosiglitazone were integrated with the microneedle array and they were subcutaneously injected into mice in a safe and effective manner. The subcutaneous adipose tissues were successfully converted into brown (beige) adipocytes, which is associated with increased energy expenditure, fatty acid oxidation, and improved insulin sensitivity. This technique is expected to allow clinical delivery of a chemical cocktail to locally induce direct reprogramming and to minimize side effects in other organs compared with oral and intravenous administrations.

## Cardiomyocytes

Ieda et al. [10] first reported that cardiac fibroblasts that constituted over 50% of the cells in the heart were directly converted into cardiomyocyte-like cells by forced expression of a set of cardiac transcription factors, *Gata4*, *Mef2c*, and *Tbx5*. After the report, modified combinations of transcription factors and miRNAs converted mouse and human dermal fibroblasts into cardiomyocyte-like cells [56–58]. However, their conversion efficiencies appear not to be sufficient for clinical purposes. In an effort to improve the conversion efficiency, a number of small molecules and growth factors have been identified. A combination of FGF2, FGF10, and vascular EGF (VEGF) promoted the cardiac reprogramming of MEFs under serum-free defined medium [59–60]. In the other reports, CHIR99021, SB431542, Forskolin, Parnate, and Y-27632, which function as WNT signaling activator, TGFβ signaling inhibitor, cAMP inducer, lysine-specific demethylase 1 (LSD1) inhibitor, and Rho-associated kinase inhibitor (ROCK), respectively, promoted the direct reprogramming in combination with the cardiac transcription factors [61,62]. Such information obtained from the study on transcription factor-based direct reprogramming is greatly useful for the development of the chemical compound-based conversion into cardiomyocytes.

The transplantation of induced cardiomyocytes is a promising approach to retrieve cardiac functions if the loss of native cardiomyocytes is the fundamental pathology, e.g. cardiac infarction. For the clinical application, forced expression of exogenous genes by virus vectors should be avoided to reduce safety concerns. Chemical compounds potentially replace the effects of transcription factors in cardiac direct reprogramming. In 2015, Fu et al. [63] reported that MEFs were converted into cardiomyocyte-like cells by a combination of six chemical compounds (CRFVPT) including CHIR99021, RepSox, Forskolin, VPA, Parnate, and TTNPB. They unexpectedly found spontaneously contracting cells after the treatment with the chemical cocktail that had been already reported to reprogram MEFs into pluripotent stem cells [64]. The beating cells and clusters appeared 6 days after the treatment. Then for the maturation, first medium including the compounds was replaced with the cardiomyocyte maintaining medium supplemented with 2i (CHIR99021 and PD0325901) and leukemia inhibitory factor (LIF), whose combination was often used to keep a pluripotent state in mouse pluripotent stem cells. Amongst the compounds, CHIR99021, RepSox, Forskolin, and VPA were most critical for the cardiac reprogramming, indicating that the activation of the WNT signaling pathway and cellular cAMP-PKA pathway in combination with the inhibition of TGFβ signaling pathway is required. VPA functions as a histone deacetylase (HDAC) inhibitor, suggesting that the activation of gene expression through epigenetic changes further supports the cardiac direct reprogramming.

A combination of seven chemical compounds, CHIR99021, A8301, BIX01294, AS8351, SC1, Y-27632, and OAC2, successfully converted human foreskin fibroblasts into cardiomyocyte-like cells [65]. The authors also found that two inhibitors of platelet-derived growth factor receptors (PDGFRs), SU16F and JNJ10198409, enhanced the conversion efficiency by repressing fibroblast specific gene expression. After generation of spontaneously beating cells, the cells were further required to undergo a maturation step with the conditioned medium that was incubated with purified cardiomyocytes differentiated from human pluripotent stem cells. Without the conditioned medium, the beating cells did not strongly induce the expression of cardiomyocyte-specific genes such as *Tnnt2* and *Nkx2.5*. Since the conditioned medium includes unknown factors secreted from the pluripotent stem cell-derived cardiomyocytes, the next step is to replace it with more defined medium containing chemical compounds and/or growth factors, particularly for a large-scale preparation of chemically induced cardiomyocytes for the transplantation therapy. In addition, several kinds of human adult fibroblasts with different ages, genders, and races need to be efficiently converted into the cardiomyocytes for the clinical use. There might be room for increase in the conversion efficiency by optimizing combinations of chemical compounds and experimental procedures.

So far these two groups have succeeded in the complete chemical conversion of MEFs and human fibroblasts into cardiomyocytes [64,65]. CHIR99021, GSK3β inhibitor, and RepSox or A83-01, TGFβ signaling inhibitors, were compounds common to the studies, suggesting that WNT activation and TGFβ inhibition are key pathways for the cardiac reprogramming. Forskolin was essential for the conversion of MEFs but for human fibroblasts Forskolin is not necessary, which might reflect a difference between species or culture conditions. The chemical cardiac reprogramming of human fibroblasts provides more insights into the molecular mechanism underlying the direct conversion. β-catenin and Smad1, which are major effectors in the downstream of WNT and BMP signaling pathways, respectively, are recruited to the promoter regions of heart developmental genes such as *Mesp1*, *Gata4*, *Nkx2.5* [65]. Although how TGFβ inhibitors affect the BMP signaling pathway and the activity of Smad protein complex with Smad1 is unclear, these results suggest that the downstream effectors of specific signaling pathways play a critical role in the cardiac direct reprogramming. In addition, the treatment with the chemical cocktail enhanced active chromatin marks, H3K4me3 and H3K27ac, and a decreased repressive chromatin mark, H3K27me3. The early molecular events were likely related to enhanced accessibility of β-catenin and Smad1 to the heart developmental genes. According to the observation, epigenetic modulators, Bix01294 and AS8351, also play a critical role in the cardiac reprogramming because the loss of one of these compounds strongly reduced the reprogramming efficiency. In the case of MEFs, both cardiac precursor and lineage markers are immediately activated after the treatment with the chemicals, while in the case of human fibroblasts most of these cardiac markers are activated after the treatment with the conditioned medium of pluripotent stem cell-derived cardiomyocytes. In both cases, the fibroblasts were first converted into beating clusters by the chemical treatments, then a different step was required for the maturation. Further studies are required to improve the experimental protocol for efficient and large-scale preparation of the cardiomyocytes and to expand availability of the chemical cocktail in human adult dermal fibroblasts isolated from patients with various backgrounds.

## Hepatocytes and pancreatic β cells

In chronic liver diseases such as hepatic cirrhosis and cancer, severely damaged hepatocytes cannot regenerate. Due to limited source of organ donors, cell transplantation therapy is a promising approach to treat such serious liver diseases by repopulating functional hepatocytes. So far, many trials have been made to generate induced hepatocytes from other cell sources such as ES/iPS cells, MSCs, and fibroblasts by forced expression of transcription factors as well as treatment with growth factors and small molecules [66–68]. Sekiya and Suzuki [11] first reported that hepatocytes can be converted from MEFs by forced expression of two transcription factors, Hnf4α and either Foxa1, Foxa2, or Foxa3. Modified combinations of transcription factors have been reported in human dermal fibroblasts [69,70]. The induced human hepatocytes were engrafted in immunodeficient mouse livers and restored the hepatic functions. Although hepatocytes without the use of transgene are desirable for the clinical application, the direct conversion from fibroblasts by chemical compounds only has not yet been reported.

Accumulating evidence has suggested that several chemical compounds play a pivotal role in hepatic differentiation and promotion of the direct reprogramming. Dexamethasone, an agonist for glucocorticoid receptor (GR), and nicotinamide are widely used for hepatic differentiation, maturation, and proliferation. One of the bile acids, tauroursodeoxycholic acid, enhanced hepatic differentiation of rat MSCs. Tauroursodeoxycholic acid functions through Farnesoid X receptor (FXR) and transmembrane G-protein-coupled bile acid receptor 5 (Table 4) [67]. Recently forced expression of Hnf1α in combination with CHIR99021 and A83-01 directly converted MEFs into hepatocyte-like cells [71]. These compounds promoted mesenchymal to epithelial transition (MET) which is required for the conversion from fibroblasts into induced hepatocytes. More recently, forced expression of either Foxa1, Foxa2, or Foxa3 in combination with seven chemical compounds, VPA, CHIR99021, RepSox, Forskolin, Parnate, DZNep, and TTNPB converted MEFs into hepatocytes [72]. These two reports suggest that combinations of chemical compounds could replace the effects of either Hnf1α or Foxa1–3, which might lead to further development of the hepatic reprogramming by chemical compounds only. The chemical approach for the hepatic reprogramming is promising for facilitating the transplantation for the treatment of chronic liver diseases.

**Table 4:** Direct reprogramming into hepatocyte-like cells by chemical compounds and exogenous gene induction

Target cell type	Source cells	Generation	Gene induction and chemical cocktail	Induction/maturation medium	References
Hepatocyte	Rat bone-marrow derived MSCs	Adult	Tauroursodeoxycholic acid	The IMDM-based medium with tauroursodeoxycholic acid for several weeks	[67]
Hepatocyte	MEFs	Fetal	Hnf1α and CHIR99021, A83-01	After infection of retrovirus expressing Hnf1α, the hepatocyte culture medium with the compounds was incubated for 5 weeks	[71]
Hepatocyte	MEFs	Fetal	Foxa1, Foxa2, or Foxa3, and VPA, CHIR99021, RepSox, Forskolin, Parnate, DZNep, TTNPB	After infection with retrovirus expressing either Foxa1, Foxa2, or Foxa3, the hepatic reprogramming medium (HRM) with the chemical cocktail was incubated for 15 days. The hepatocyte maintaining medium (HMM) with the chemical cocktail for further culture	[72]

The number of patients suffering from diabetes mellitus has been rapidly expanding all over the world, but the fundamental treatment is difficult without transplantation of islet or functional β cells. So the development of induced pancreatic β cells from other somatic cells is one of the most attractive strategies to treat diabetes. A number of studies have reported that functional β cells were transdifferentiated from pancreatic exocrine cells, pancreatic ductal cells, intestinal crypt cells, stomach cells, and hepatocytes by forced expression of β cell lineage-specific transcription factors such as *Pdx1*, *Ngn3*, and *MafA* [73–76]. These studies utilized developmentally relevant cells such as pancreatic cells and other endoderm lineage cells, suggesting that the starting cell types are critical for the direct reprogramming into β cells. Corresponding to the complicated developmental pathway of pancreatic β cells *in vivo*, proper differentiation from pluripotent stem cells requires many kinds of culture media, growth factors, and small molecules at each step of the differentiation [77,78].

In consideration of these background factors, chemical compound-based direct conversion of human dermal fibroblasts into functional β cells is challenging. In practice, such chemical conversion into β cells has not yet been reported. Sheng Ding and colleagues demonstrated that transient expression of the reprogramming factors such as Oct4, Sox2, Klf4, and c-Myc or P53 shRNA converted mouse and human fibroblasts into an intermediate [79,80]. Then, many steps using various combinations of chemical compounds and growth factors converted the intermediate into β-like cells via definitive endodermal progenitor cells and pancreatic endodermal progenitor cells. Now it is unclear whether it is feasible to directly convert human dermal fibroblasts into functional β cells by using a specific set of chemical compounds. However, such a chemical strategy is worthwhile for trying to rapidly prepare functional β cells from autologous fibroblasts for the transplantation to patients suffering from type I and type II diabetes.

The high-throughput screening of small molecules from several groups have identified candidate compounds for chemical compound-based pancreatic reprogramming. BRD7389, an inhibitor of ribosomal S6 kinase (RSK), changed the cell shape of mouse pancreatic α cells to a more β cell-like one and increased the expression of β cell-specific genes, *Ins2* and *Pdx1* [81]. BRD7552 was identified amongst 60000 compounds using PANC-1 cell, a human pancreatic ductal carcinoma cell line [82]. BRD7552 increased the expression of *Pdx1* gene in both primary human islets and the ductal cells. 5′-Azadeoxycytidine (AZA), a DNA methyltransferase inhibitor, induced *Ngn3* expression in PANC-1 cells and promoted an endocrine differentiation in the cells [83]. Harmine induced proliferation of murine and human β cells through inhibition of dual-specificity tyrosine-regulated kinase-1a (DYRK1A) [84]. More recently, one of the screened small molecules, AT7867, induced proliferation of Pdx1-positive pancreatic progenitor cells differentiated from human iPS cells [85].

## Endoderm progenitor cells and somatic stem cells

Challenges to converting either differentiated endoderm cells or fibroblasts into the progenitor cells have been noted by several groups. Wang et al. [86] reported that human gastric epithelial cells (hGECs) isolated from the stomach were chemically converted into endoderm progenitor cells with tissue-specific mesenchymal feeder cells (Table 5). The human induced endoderm progenitor cells (hiEndoPCs) were proliferative and expressed endoderm markers, Sox17 and Foxa2. hiEndoPCs were subsequently differentiated into hepatocytes, pancreatic endocrine cells, and intestinal epithelial cells. The conversion requires human gastric subepithelial myofibroblasts (GSEMFs) as feeder cells, and a surgery to prepare these hGECs and GSEMFs from the stomach. Such partial reprogramming of fully differentiated endoderm cells is promising for preparing other important endoderm cells such as hepatocytes and β cells, which could be applied for transplantation therapy to treat chronic liver diseases and diabetes.

**Table 5:** Summary of chemical compound-based direct reprogramming into endoderm progenitor cells and somatic stem cells

Target cell type	Source cells	Generation	Chemical cocktail cytokines	Induction/maturation medium	References
Endoderm progenitor cells	hGECs	Adult (35–78 years old)	Bix01294, RG108, Bay k 8644, and SB431542	The DMEM-F12 based medium with the chemical cocktail using human GSEMFs (hGSEMFs) as feeder cells for 7–15 days. The DMEM-F12 based medium with A83-01, Wnt3a, and bFGF for further culture and passage	[86]
Endoderm progenitor cells	MEFs	Fetal	RepSox, Forskolin, Y-27632, CHIR99021, TTNPB, bFGF, BMP4, and Activin A	The N2B27 medium with the chemical cocktail including TTNPB for 4 days (Stage 1). The N2B27 medium with the chemical cocktail including high concentration of CHIR99021 and Activin A instead of TTNPB for another 10 days (Stage 2). The modified medium mixed with the commercial HCM medium for further culture (Stage 3).	[87]
Chemically induced liver progenitors (CLiPs)	Rodent primary hepatocytes	Adult	Y-27632, A83-01, and CHIR99021	Rodent primary hepatocytes under the culture treated with the three chemicals become proliferative without obvious phenotypic alterations.	[88]
MSC	Human dermal fibroblasts	Fetal adult	SP600125, SB202190, GO6983, Y-27632, PD0325901, and CHIR99021	The chemical cocktail medium with the six chemicals and TGF-β1, bFGF, and LIF for 6 days. After FACS sorting, the MSC expansion medium for further culture and expansion	[91]

Recently MEFs were chemically converted into endoderm progenitor cells by two sets of combinations of chemical compounds and growth factors [87]. MEFs were first treated with RepSox, Forskolin, Y-27632, a low concentration of CHIR99021, TTNPB, and two kinds of cytokines, bFGF and BMP4. In the second step, a high concentration of CHIR99021 and Activin A were added to the first chemical cocktail without TTNPB. The chemically induced endoderm progenitor cells (ciEPCs) formed colonies, and they were stably expanded over 13 passages. ciEPCs expressed endoderm marker genes such as *Cdh1*, *Sox17*, *FoxoA2*, and *HNF4α*. ciEPCs could be differentiated into hepatocyte-like cells in the specialized medium including chemical compounds and growth factors, DAPT, Dexamethasone, A83-01, hepatic growth factor (HGF), and oncostatin M (OSM). The hepatocyte-like cells differentiated from ciEPCs exhibited an expression pattern similar to the one of iPS cells-derived hepatocytes. ciEPCs were also differentiated into pancreatic progenitor cells expressing Pdx1; however, insulin expression was not confirmed. It is also a promising strategy to prepare clinically applicable hepatocytes and pancreatic β cells if human adult fibroblasts are successfully converted into the endoderm progenitor cells as well. Similar combinations of the chemicals and the protocols might be useful for performing the conversion of human fibroblasts into ciEPCs.

Katsuda et al*.* [88] successfully generated expandable hepatocyte progenitor cells by culturing rodent primary hepatocytes under the treatment with three chemicals, Y-27632, A83-01, and CHIR99021. The chemically induced liver progenitors (CLiPs) were stably proliferative under the chemical cocktail without any phenotypic alterations. CLiPs were bipotent and could be differentiated into both mature hepatocytes and biliary epithelial cells. In addition, CLiPs repopulated chronically injured liver tissues at a high rate (75–90%). The next step for the clinical application is to develop CLiPs from human primary hepatocytes; however, human CLiPs have not been generated with the same compounds, which is possibly due to difference in the molecular mechanisms between human and rodent [89]. A different set of chemical compounds might be required. The transplantation of CLiPs is a promising therapeutic strategy for liver regenerative medicine.

MSCs are multipotent somatic stem cells and can be differentiated into osteocytes, chondrocytes, and adipocytes. MSCs are generally isolated from the tissues such as bone marrow, adipose tissue, and umbilical cord blood. MSCs also have immunomodulatory effects to suppress immune response and participate in tissue repair by secreting multiple cytokines [90]. Human dermal fibroblasts were chemically converted into MSC-like cells at approximately 38% in just 6 days [91]. The chemical cocktail contains six compounds, SP600125, SB202190, GO6983, Y-27632, PD0325901, and CHIR99021, in combination with three growth factors, TGFβ1, bFGF, and LIF. The induced MSCs had a higher proliferation rate than fibroblasts, which was maintained for at least eight passages. The induced MSCs were differentiated into osteoblasts, adipocytes, and chondrocytes, similar to the multipotent capacity in MSCs isolated from human tissues. This technique is obviously useful to generate expandable MSCs from dermal fibroblasts isolated from each patient. The induced MSCs present little safety concern about tumorigenesis, so transplantation therapy using the induced MSCs themselves and differentiated osteocytes and chondrocytes might be available for future regenerative medicine.

## Pluripotent stem cells

In 2013, Hou et al. [64] first reported that pluripotent stem cells were chemically reprogrammed from MEFs without any induction of transcription factors (Table 6). The treatment with a chemical cocktail containing seven compounds, VPA, CHIR99021, E-616452 (also named as RepSox), Tranylcypromine, Forskolin, DZNep, and TTNPB, generated chemically induced pluripotent stem cells (CiPSCs). They first identified that the effects of expression of Oct4, one of the reprogramming factors, could be obtained with substitution with Forskolin. Their group has already reported that the combination of VC6T (VPA, CHIR99021, E-616452, Tranylcypromine) in addition to forced expression of Oct4 could replace the effects of other reprogramming factors and reprogrammed MEFs toward iPS cells [15]. Therefore Forskolin, a potential substituent for Oct4, was combined with the VC6T. To achieve complete reprogramming into CiPSCs, DZNep was further added 16 days after the treatment with VC6TF. The generated CiPSCs were integrated with early embryos, and successfully generated viable chimera mice, suggesting that CiPSCs have a differentiation potential similar to iPS cells and ES cells.

**Table 6:** Summary of chemical compound-based reprogramming into induced pluripotent stem cells

Target cell type	Source cells	Generation	Chemical cocktail cytokines	Induction/maturation medium	References
Induced pluripotent stem cells	MEFs	Fetal	VC6TF: VPA, CHIR99021, E-616452, Tranylcypromine, and Forskolin VC6TFZ: VC6TF and DZNep	The chemical reprogramming medium with the chemical cocktail (-DZNep) for 16–20 days, followed by the medium with the chemical cocktail (+DZNep) for another 12–16 days. The medium supplemented with 2i, CHIR99021 and PD0325901, and LIF for further culture	[64]
Induced pluripotent stem cells	MEFs	Fetal	VC6TFAE: VPA, CHIR99021, E-616452, Tranylcypromine, Forskolin, AM580, and EPZ004777 VC6TFZASD: VC6TFZA and SGC0946, 5-Aza-dC	The chemical reprogramming medium with the chemical cocktail, VC6TFAE, for 16 days, followed by the medium with the chemical cocktail, VC6TFZASD, for another 12 days. The N2B27 medium with 2i (CHIR99021 and PD0325901) and LIF for another 12 days	[92]
Induced pluripotent stem cells	Mouse neural stem cells	Fetal and postnatal	VC6TFE5: VPA, CHIR99021, E-616452, Tranylcypromine, Forskolin, EPZ004777, and Ch55 VC6TFE5Z: VC6TFE5 and DZNep	The chemical reprogramming medium with the chemical cocktail, VC6TFE5 (E-616452: 2 or 5 μM), for 20 days, followed by the medium with the chemical cocktail, VC6TFE5Z (E-616452: 5 μM), for another 20 days. The N2B27 medium with 2i CHIR99021 and PD0325901 for further culture	[93]
Induced pluripotent stem cells	Mouse small intestinal epithelial cells	Fetal	VC6TFA: VPA, CHIR99021, E-616452, Tranylcypromine, Forskolin, and AM580 VC6TFZ: VC6TF and DZNep	The chemical reprogramming medium with the chemical cocktail, VC6TFA (E-616452: 20 μM), for 16 days, followed by the medium with the chemical cocktail, VC6TFZ (E-616452: 10 μM), for another 24 days. The N2B27 medium with 2i, CHIR99021 and PD0325901, for further culture	[93]

Approximately 2 years later, the same group showed that extraembryonic endoderm (XEN)-like cells were an intermediate in the process of CiPSC generation [92]. The XEN-like cells strongly expressed two master genes for pluripotency, *Sall4* and *Lin28a*, which makes the pluripotent state more accessible. The transition via XEN-like cells in the reprogramming process is unique for chemical-based, but not observed in transcription factor-based, reprogramming. In addition, the effects of several new compounds such as AM580, EPZ004777, SGC0946, and 5-Aza-dC on the chemical reprogramming were investigated. The retinoic acid receptor agonist and epigenetic modulators in combination with VC6TF increased the efficiency up to 1000-fold greater than that described in their previous reports. Not only MEFs but also neonatal dermal fibroblasts and adult lung fibroblasts were chemically reprogrammed, suggesting a broad availability of the chemical cocktail for the reprogramming. Further studies are required to reveal in more detail the molecular mechanism by which each chemical compound facilitates the chemical reprogramming through regulation of targetted signaling pathways and epigenetic modifications. The identification of an intermediate state is an important step to further improve the experimental protocol and the combination of chemical compounds for rapid and better reprogramming to a pluripotent state that resembles that of ES cells.

Moreover, their group also successfully converted mouse neural stem cells and small intestinal epithelial cells into CiPSCs [93]. Similar but slightly different combinations of the chemical compounds were required in these cell types, suggesting that a similar molecular mechanism underlying the chemical reprogramming might be conserved amongst diverse cell types. Although the combination of the core chemicals (VC6TF: VPA, CHIR99021, RepSox, Tranylcypromine, and Forskolin) was not changed, fine-tuning of the concentrations and treatment period was required in the chemical reprogramming of different cell types. In particular, RepSox, a potential substituent for Sox2 overexpression, is likely less required in the neural stem cells than the intestinal epithelial cells, probably because Sox2 is already expressed at a high level in the neural stem cells. The CiPSCs derived from the different cell types contributed to mouse chimera formation. Another report suggested that the chemically induced XEN-like cells were expandable without any genome instability and were directly converted into functional neurones and hepatocytes [94]. The XEN-like state provides an alternative strategy to prepare desired cell types by bypassing a pluripotent state.

CiPSCs might have higher developmental potentials than iPSCs induced by the reprogramming transcription factors [64,93,95]. CiPSCs might more efficiently contribute to chimera formation than the conventional iPSCs. Genome-wide analysis of DNA methylation revealed that CiPSCs were more hypomethylated than the conventional iPSCs, particularly in several regions of imprinted gene clusters [96]. The authors suggested that the hypomethylation status of CiPSC was closer to that of mouse ES cells, which might explain the higher developmental potentials of CiPSCs.

Because human somatic cells have not been reprogrammed into CiPSCs by chemical compounds only, the generation will have a great impact on this field. Several differences of signaling pathways between mouse and human pluripotent stem cells have been reported [97,98]. One of the most characteristic differences is that mouse ES cells maintain a naïve pluripotent state under the 2i (CHIR99021 and PD0325901) and LIF condition, while modification of multiple signaling pathways by chemical compounds was required to maintain such naïve pluripotency in human ES cells [99]. Therefore, the molecular mechanism underlying the chemical reprogramming into human CiPSCs might be more complicated than and different from that in the mouse. Possibly, the reprogramming requires a different set of chemical compounds. The chemical compound-based reprogramming in human somatic cells will be worth pursuing if CiPSCs have clear advantages in terms of safety, developmental potentials, and the derivation process.

## Chemical compounds for direct reprogramming

In most cases, the combination of chemical compounds required for each direct conversion were empirically determined, and the detailed molecular mechanisms of the direct conversions remain largely unknown. However, identification of the chemical compounds provides great insights into how signaling pathways regulate the direct conversion. In addition, we need to understand how multiple signaling pathways could synergistically change one cell fate to another. A better understanding of the reprogramming process is obviously helpful to improve the efficiency and to perform complete epigenetic reprogramming into various cell types which are more similar to the *in vivo* ones.

A number of common chemicals are frequently used in the direct conversions from both mouse and human fibroblasts. CHIR99021 is one of the most frequently used compounds in various conversions into neurones, neural stem cells, cardiomyocytes, endoderm progenitor cells, and iPS cells, suggesting that activation of WNT signaling pathway by inhibiting GSK3 might potentially increase plasticity for cell fate changes in fibroblasts. On the other hand, our report indicated that CHIR99021 severely inhibited the brown adipogenic reprogramming, which suggests that a cell type-specific regulation of signaling pathways is required for chemical direct reprogramming [53]. TGFβ signaling pathway inhibitors such as SB431542, RepSox, and A83-01 were frequently used for the direct conversions into various cell types. Previous reports have suggested that the TGFβ inhibition is associated with facilitation of MET as well as suppression of profibrotic signals [100,101]. ROCK inhibitors such as Y-27632 and Thiazovivin were also used in the conversions into neurones, neural stem cells, cardiomyocytes, and endoderm and liver progenitor cells. A recent report demonstrated that ROCK inhibitor ameliorated cellular senescence and improved mitochondrial function by promoting metabolic reprogramming from glycolysis to oxidative phosphorylation in human fibroblasts [102]. In addition, the ROCK inhibition activated chromatin remodeling genes, which might be associated with a change in chromatin structures and epigenetic modifications. As shown in the cardiac direct reprogramming by transcription factors and small molecules [101], the inhibition of ROCK and TGFβ signaling pathways led to repressed expression of cytoskeletal and extracellular matrix proteins, which can facilitate direct reprogramming into other cell types, particularly from fibroblasts.

A few articles have reported a possible molecular mechanism underlying chemical direct reprogramming. A study on neural stem cell reprogramming suggested that downstream effectors, Elk1 and Gli2, in MAPK and SHH signaling pathways, respectively, were both directly bound to the promoter of *Sox2* gene and activated the expression [42]. In human cardiac reprogramming, β-catenin and Smad1 in the downstream of the WNT and BMP signaling pathways are directly recruited to cardiac developmental genes [65]. In addition, the chemical cocktail required for the cardiac reprogramming opened a heterochromatin state into a euchromatin state, leading to enhanced chromatin accessibility of the downstream transcription factors. In both cases, the regulation of specific signaling pathways by chemical compounds could endogenously activate key developmental genes, leading to establishment of the cell type-specific transcriptional network. The combination of simultaneous activation of chromatin and specific signaling pathways is likely required to accomplish direct reprogramming. At the moment, this could be one of the most rational explanations for the molecular mechanism underlying complete chemical direct reprogramming.

One of the characteristics in chemically induced cells is low tumorigenicity, although whether it is really lower than that for the cells converted by transcription factors remains unknown. More careful studies will be required to evaluate the tumorigenicity in chemically induced cells by transplantation into animal models. The treatment with chemical compounds likely changes broad gene expression via downstream transcription factors of the related signaling pathways, which might also lead to an irreversible disruption of proper epigenetic states. It might increase the risk for tumorigenesis although this should be dependent on the properties of chemicals, concentration, incubation time, and so on.

Compared with gene expression regulated by specific transcription factors, chemical compounds do not always activate expression of a specific set of transcription factors required for the cell fate conversion and have an influence on a wide range of gene expression and epigenetic modifications. Therefore, to more specifically and selectively regulate signaling pathways and histone-modifying enzymes, the strategy of chemical biology is required to identify novel compounds from amongst a tremendous array of chemicals. Such novel compounds might improve on the direct reprogramming efficiency, and lead to the conversions into more kinds of cell types that have not yet been reported.

## Conclusion

This review provides an overview of recent advances in chemical compound-based direct reprogramming. This field has rapidly developed based upon numerous studies on transcription factor-based direct reprogramming. The combinations of chemical compounds and the cell type-specific medium including growth factors will likely enable more cell fate conversions of somatic cells in the future. The use of chemical compounds instead of forced expression of transcription factors resolves several barriers which have been raised in the use of iPS cells and cells directly reprogrammed by transcription factors. Such small molecule-based direct conversions in a chemically defined medium will robustly facilitate the cell-based study on transplantation therapy, disease modeling, drug screening, and personalized medicine (Figure 3).

Further studies are required to more fully manipulate cell fates by chemical compounds only. In addition, most reported protocols for chemical compound-based direct reprogramming are performed and repeated by the same groups. To increase the availability in other laboratories as well as hospitals, the same or similar experiments need to be actively repeated by independent groups. Particularly, the chemical conversion of human fibroblasts into endoderm lineage cells such as hepatocytes and pancreatic β cells is clinically anticipated. The chemical compound-based strategy could become one of the main sources for cell transplantation therapy in the near future.

## Abbreviations

BAM: Brn2, Ascl1, and Myt1
BDNF: brain-derived neurotrophic factor
Bex: bexarotene
bFGF: basic fibroblast growth factor
BMP: bone morphogenetic protein
ciBA: chemical compound-induced brown adipocyte
ciEPC: chemically induced endoderm progenitor cell
CiNC: chemical compound-induced neuronal cell
CiPSC: chemically induced pluripotent stem cell
CLiP: chemically induced liver progenitor
EGF: epidermal growth factor
ESCs: embryonic stem cells
FGF2: fibroblast growth factor 2
GABA: gamma-amino butyric acid
GDNF: glial cell line-derived neurotrophic factor
GSEMF: gastric subepithelial myofibroblast
GSK3β: glycogen synthase kinase 3β
H3K4me3: histone 3 lysine 4 trimethylation
H3K27ac: histone 3 lysine 27 aectylation
H3K27me3: histone 3 lysine 27 trimethylation
hGEC: human gastric epithelial cell
hiEndoPC: human induced endoderm progenitor cell
LIF: leukemia inhibitory factor
MAPK: mitogen-activated protein kinase
MEF: mouse embryonic fibroblast
MET: mesenchymal to epithelial transition
MSC: mesenchymal stem cell
NPC: neural progenitor cell
NT3: Neurotrophin 3
PKA: protein kinase A
ROCK: Rho-associated kinase inhibitor
RXR: retinoid X receptor
SHH: sonic hedgehog
TGFβ: transforming growth factor β
XEN: extraembryonic endoderm
